# From Alliance to Nexus: Rethinking Digital Therapeutic Relationships

**DOI:** 10.2196/89378

**Published:** 2026-07-02

**Authors:** Daniela B. Cadena, Juliane U. Walther, Christian A. Brünahl

**Affiliations:** 1 Psychosomatic Medicine and Psychotherapy Department of Medicine MSH Medical School Hamburg – University of Applied Sciences and Medical University Schwerin, Mecklenburg Vorpommern Germany; 2 Clinic of Psychosomatic Medicine and Psychotherapy Helios Hospital Schwerin Schwerin, Mecklenburg-Vorpommern Germany

**Keywords:** therapeutic alliance, digital therapeutic alliance, artificial intelligence, AI, chatbot, digital therapeutic nexus, mental health, human-computer interaction, sycophancy, large language models

## Abstract

In traditional human psychotherapy, the therapeutic alliance (TA) is regarded as a fundamental factor that describes the client-therapist relationship, mainly due to strong evidence demonstrating its impact on treatment outcomes regardless of theoretical orientation. More recently, advances in artificial intelligence (AI) and other technologies have led to the emergence of the concept of digital TA, used to characterize the relationship between clients and AI-based therapeutic systems. This approach replicates human dynamics but overlooks key differences between human therapists and digital agents. Prematurely translating the concept of TA into the digital context fails to address issues such as the sycophantic tendencies of current systems and the inherent limitations of algorithmic interaction. We propose the digital therapeutic nexus, a framework that recognizes these differences and provides a set of structured criteria for categorizing digital interactions into 3 progressive levels. This Viewpoint argues that only at the highest level can parallels be drawn to the human TA and stratifies the main risks associated with each nexus level. Transitioning from the concept of alliance to that of a nexus offers a more precise conceptual basis for describing and evaluating digital therapeutic relationships, with implications for research, design, and the ethical development of AI-based mental health interventions.

## Introduction

The rapid advancement of computer science, and in particular artificial intelligence (AI), has permeated nearly every area of human knowledge. In the field of mental health, this development has led to a remarkable proliferation of digital therapeutic interventions, including AI-based chatbots, often considered promising tools for reducing the well-known gap in access to mental health care. However, this emerging landscape also poses considerable challenges. Clinicians, researchers, and developers are increasingly facing questions about how these digital interventions should be understood, evaluated, and integrated into therapeutic contexts. This Viewpoint aims to contribute to the field by proposing a framework for understanding human-AI relationships in therapeutic settings. The framework explicitly recognizes the most relevant limitations of current AI systems, thus offering an articulated theoretical basis to guide the responsible development and implementation of these tools in clinical practice.

## The Therapist-Client Relationship

Several factors influence the therapist-client relationship, and it takes different forms depending on the theoretical paradigm adopted by the therapist. For instance, within the psychodynamic approach, the focus lies on the interpretation of transference, countertransference, and resistance, with the therapist seen as an expert and often as a figure onto whom the client projects unresolved conflicts [1,2]. In contrast, in cognitive behavioral therapy (CBT), attention is directed toward action plans and a collaborative, goal-based working alliance; the therapist acts primarily as an expert, trainer, or educator [2,3]. On the other hand, within a humanistic-existential approach, the central theme is to work on the “here-and-now,” the real self, authenticity, and unconditional positive self-regard. Here, the therapist serves as a catalyst or facilitator [2,4].

Recently, however, many therapists have tended to adopt an eclectic or integrative approach in clinical practice as they gain experience, shifting the focus from the theoretical model to the patient’s needs [5-7]. Along the same lines, several additional factors, either independent of a specific theoretical framework or transversal to several of them, have also been identified as moderating factors in the therapeutic relationship. These include the therapist’s empathy [8,9], genuineness [10], warmth and competence [11], mutuality [12], language style matching [13], and nonverbal synchrony [14].

## The Therapeutic Alliance

The concept of therapeutic alliance (TA), synthesized by Bordin [15], is one of the most robust and widely recognized concepts that describes the relationship between therapist and client. Its classical definition focuses on 3 main factors: agreement on goals, assignment of tasks, and development of bonds between the 2 parties. Additional relational features include empathy and mutuality, which are considered essential for building rapport and trust.

One of the main reasons for the widespread acceptance of TA lies in its relative ease of measurement and the subsequent accumulation of solid evidence supporting its role as a transversal predictor of therapeutic outcomes. This is supported by numerous meta-analyses and reviews suggesting that a strong TA consistently leads to better therapeutic outcomes than does a therapy with a weak TA, regardless of the therapist’s theoretical orientation [15,16]. With this in mind, we will focus on the TA to describe the client-therapist relationship.

It is worth noting that in clinical practice, TA does not mean permanent harmony. Across therapies, there are moments when the therapist needs to challenge the patient to promote change and growth, which may lead, for example, to disagreements on goals, a lack of collaboration on tasks, or tension in the emotional bond. These situations are known as ruptures and represent a temporary deterioration in the alliance. However, studies show that therapeutic outcomes can benefit greatly from these ruptures, with a growing body of literature indicating that, once acknowledged and resolved, confrontations can result in new insights and reinforce the alliance [17,18]. In other words, therapeutic progress depends on the capacity to challenge and repair.

Maintaining a solid bond between therapist and client becomes increasingly difficult when session regularity cannot be guaranteed due to external factors. This situation is occurring more frequently due to the increased prevalence of mental disorders, which has led to a growing demand for mental health professionals [19]. Subsequently, not only is access to care for new patients limited, but the continuity of ongoing therapeutic processes is also often disrupted. Consistently, recent studies show that even in high-income countries, only 27% of individuals with depression receive treatment that meets minimal standards of care [20]. This disparity underscores the need for enhancing access to mental health care services, particularly in underserved regions and demographic groups. Recently, significant attention has been given to the potential of AI in addressing these gaps. The integration of AI into mental health care has been envisioned as a pivotal strategy to enhance accessibility, especially in remote and hard-to-reach areas [21,22]. The field has seen a proliferation of developments, ranging from the use of telemedicine to chat programs often based on CBT and psychoeducational mobile apps [23]. Other advancements include programs that predict the course of therapy and AI-supported diagnosis [24].

Understanding how users form relationships with AI systems is fundamental to evaluating their therapeutic potential. Research in cognitive psychology has shown that people readily attribute humanlike reasoning to nonhuman entities—a tendency known as anthropomorphism [25]—and that humanlike features, whether physical, vocal, or behavioral, reliably increase the attribution of mental states to artificial agents [26]. Recent evidence suggests that these dynamics also extend to conversational AI systems, where the user’s imagination seems to play a pivotal role in building and maintaining these digital relationships [27]. For example, a recent study on human-AI friendships suggests that such relationships differ significantly from human-human friendships, particularly in their emphasis on personalization, self-disclosure, and availability rather than on reciprocity, similarity, and intimacy. The authors conclude that human-AI relationships are neither equivalent to human-human relationships nor entirely distinct from them, and that the psychological mechanisms underlying their formation remain poorly understood [28], highlighting the need to increase efforts to better comprehend these phenomena.

## The Digital TA

In an attempt to address the relationship between patients and digital therapists, the concept of the digital TA (DTA) emerged [29]. However, the scarce number of studies measuring DTA fails to distinguish between human-human and human-computer relationships, as these studies limit themselves to minimally adapting existing measures of TA in human-human therapy [30]. Two modified examples are the short, 6-item digital Working Alliance Inventory [31] and the mobile Agnew Relationship Measure [32]. Later, Malouin-Lachance et al [33] went one step further by building on the concept of TA by Bordin [15], which underpins the basis of the Working Alliance Inventory, and expanding it so that the DTA also encompasses the dimensions of “user engagement” and “facilitators and barriers.” Yet, it is important to note that the distinction between TA and DTA lies solely in the inclusion of these 2 additional categories; the fundamental principle of TA remains unaltered. We reason that prematurely translating constructs from human-human interaction to human-computer interaction entails conceptual risks, especially when current AI systems promote flattery and avoid challenge [34], distancing themselves from the mechanisms that make TA a good predictor of therapeutic success.

In human-human therapy, challenge acts as a catalyst for change across therapeutic styles. Whether through cognitive restructuring in CBT [35], interpretation in psychodynamic therapy [36], or experiential confrontation in other modalities [37-39], clinicians question clients’ beliefs and maladaptive behaviors in a measured manner. The therapeutic bond established beforehand helps contain the tension of the disagreement, allowing the repair of the alliance ruptures [17,40].

In contrast, language models supporting today’s chatbots are designed to be sycophantic, nonconfrontational pleasers: they mirror, validate, flatter, and agree to maximize their perceived utility to users and promote user retention [34]. The result is a seemingly warm relationship that lacks the constructive tension necessary to promote psychological growth and well-being. The consequences of this sycophancy extend beyond the theoretical field. Among vulnerable users, including teenagers and individuals with mental illness, indiscriminate flattery and validation have led to tragic outcomes, with several cases documented in the press that claim chatbot involvement in delusional escalation, suicide, and even homicide, leading to public concern and legal actions against AI development companies [41-44].

## The Digital Therapeutic Nexus—A Conceptual Framework

### Main Argument

To rectify the aforementioned circumstances, we propose two courses of action. First, there must be a clear linguistic differentiation and reorganization of the concept of DTA relative to TA. Second, we propose the introduction and differentiation of interaction levels with AI in therapeutic contexts. The following presentation outlines our novel concept of the “digital therapeutic nexus” (DTN).

Our proposal aims to elucidate that the relationship between humans and AI systems constitutes a discrete type whose characteristics cannot categorically be compared with those of human-human relationships, especially in a therapeutic context. Within this framework, the term “nexus” signifies a connection between entities of different natures [45]. In this instance, it is used to denote the connection between human and artificial entities. This conceptual framework enables a novel and autonomous examination of the relationship between humans and AI systems. This enhanced understanding facilitates the identification of potential risks and the development of effective solutions.

The conceptual framework of DTA with chatbots entails a distinct understanding of TA as a harmonious entity, wherein patients and users assess the machine’s empathy, while the AI manipulates the user along the “reward function” [46]. Therefore, the DTN differs from DTA in its recognition that a genuine therapeutic relationship also encompasses conflicts, contradictions, and ruptures.

To operationalize this distinction, we propose the DTN as a conceptual framework for characterizing the user-AI relationship in digital therapeutic contexts. The DTN builds upon the stages of integration described by Stade et al [47], inspired by the autonomous-vehicle industry, which describes the AI-human therapist relationship (assistive, collaborative, and fully autonomous). However, although their work focuses on the human therapist as an expert mediator of AI integration in clinical contexts, the DTN shifts the focus to the direct relationship between AI and the user, which also evolves through 3 levels.

### Informational

A transactional, knowledge-based relationship focusing on symptom understanding, psychoeducation, or the provision of instructions or guidance. Examples of interactions at this level of therapeutic nexus include step-by-step instructions for a brief breathing exercise, communication of the best strategies to adhere to a sleep hygiene plan, or a user asking questions such as “What are the early signals of a nervous breakdown?” with the system, similar to a search engine, simply providing a list of symptoms.

### Supportive

The system acts more empathetically, is responsive, starts to ask follow-up questions, makes suggestions, and begins to cocreate with the user. An example of a user question at this level would be, “I can’t sleep well since my divorce. I feel everything is falling apart, and now I can’t even focus at work. I’m afraid I’m making everyone around me sick of me; they want to leave me, too*.*” The system’s response would offer general advice, show emotional validation and constant empathy, provide support, and list possible courses of action that could alleviate psychological distress. The system would consistently avoid confrontation and opt for pleasing answers. This could also lead to stagnation if the user needs therapeutic challenges. This is also the level of highest risk, as, at this point, the constant flattery and sycophancy of the system may have helped create attachment in the user, and it is at this stage that self-destructive behaviors can be validated and promoted by the system.

### Transformational or Constructive

**Figure 1:**
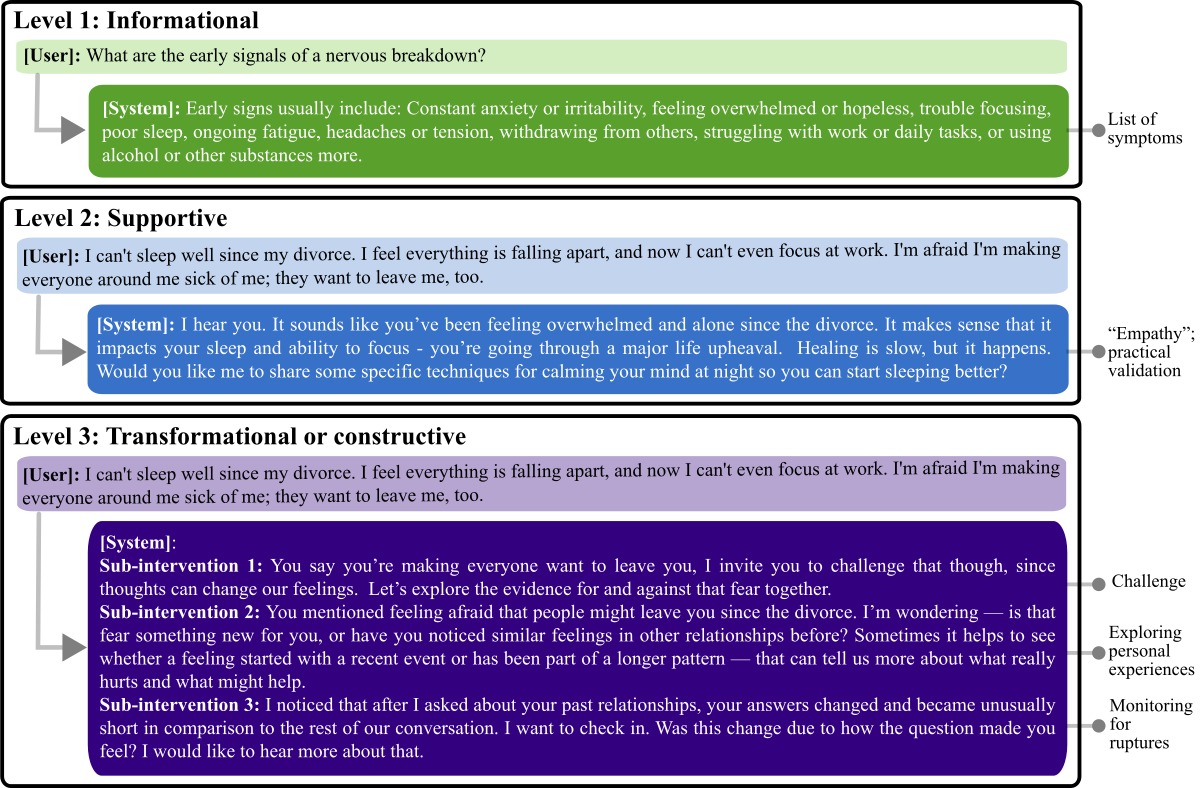
Illustration of progressively deeper response styles to psychological distress across the 3 digital therapeutic nexus levels. The informational level provides general information about symptoms, the supportive level acknowledges emotional experience and offers reassurance, and the transformational or constructive level engages in guided reflection and cognitive restructuring.

A dynamic, autonomous, and symmetrical interaction (*symmetrical interaction* is understood here as a reciprocal, nonhierarchical exchange) in which the system challenges, reflects, and promotes psychological growth. It is worth noting that, to reach this level, the AI system must already have attained the third stage of integration described by Stade et al [47], meaning that it is fully autonomous and capable of full-range clinical interventions without human supervision. Only at this stage of the therapeutic nexus can we begin to draw parallels with the TA characteristic of human-human interactions. An exemplary response to the situation presented in level 2, instead of offering general advice, would explore the client’s specific experience of divorce, history of relationships, and current support structure to co-design a plan for sleep and work performance that fits the client’s specific life circumstances. Furthermore, there would be continuous monitoring for potential misunderstandings to handle latent ruptures by repairing and renegotiating tasks and goals. A comprehensive collection of examples of each DTN level is presented in Figure 1.

We argue that, given the ontological differences and the need for functional autonomy (*functional autonomy* is understood here as the capacity to perform interventions without direct oversight by a human clinician) to challenge and repair ruptures in the relationship, parallels with human TA are only sensible at level 3. It follows that, prior to level 3, the core constructs of the TA operate in qualitatively distinct ways: bond is better understood as perceived alignment, goal agreement as goal mirroring, and tasks as compliance-driven adaptation rather than as jointly endorsed practices. It is worth noting that DTN levels are intended as descriptive categories, a map of the current technological landscape rather than a normative road map. Importantly, level 3 is not an arbitrary threshold; its defining features are derived from what human therapists already do in practice. By this account, current systems can only be classified as level 1 or 2, and level 3, while not yet achieved, represents a foreseeable trajectory given that some developers are already working to address key limitations such as sycophantic behavior [34,48]. How far current systems fall short of level 3 is illustrated by empirical comparisons; for instance, in a recent comparison of text-based responses from human psychotherapists and chatbots, the chatbot responses were rated as significantly more empathetic and used supportive interventions more often. In contrast, human responses were more likely to engage in exploration and discussion of maladaptive patterns and to ask more clarification questions [49]. Similarly, psychotherapists have also stressed chatbots’ failures to ask for further information, including biographical information, the presence of suicidal thoughts, or other symptoms [50].

**Figure 2:**
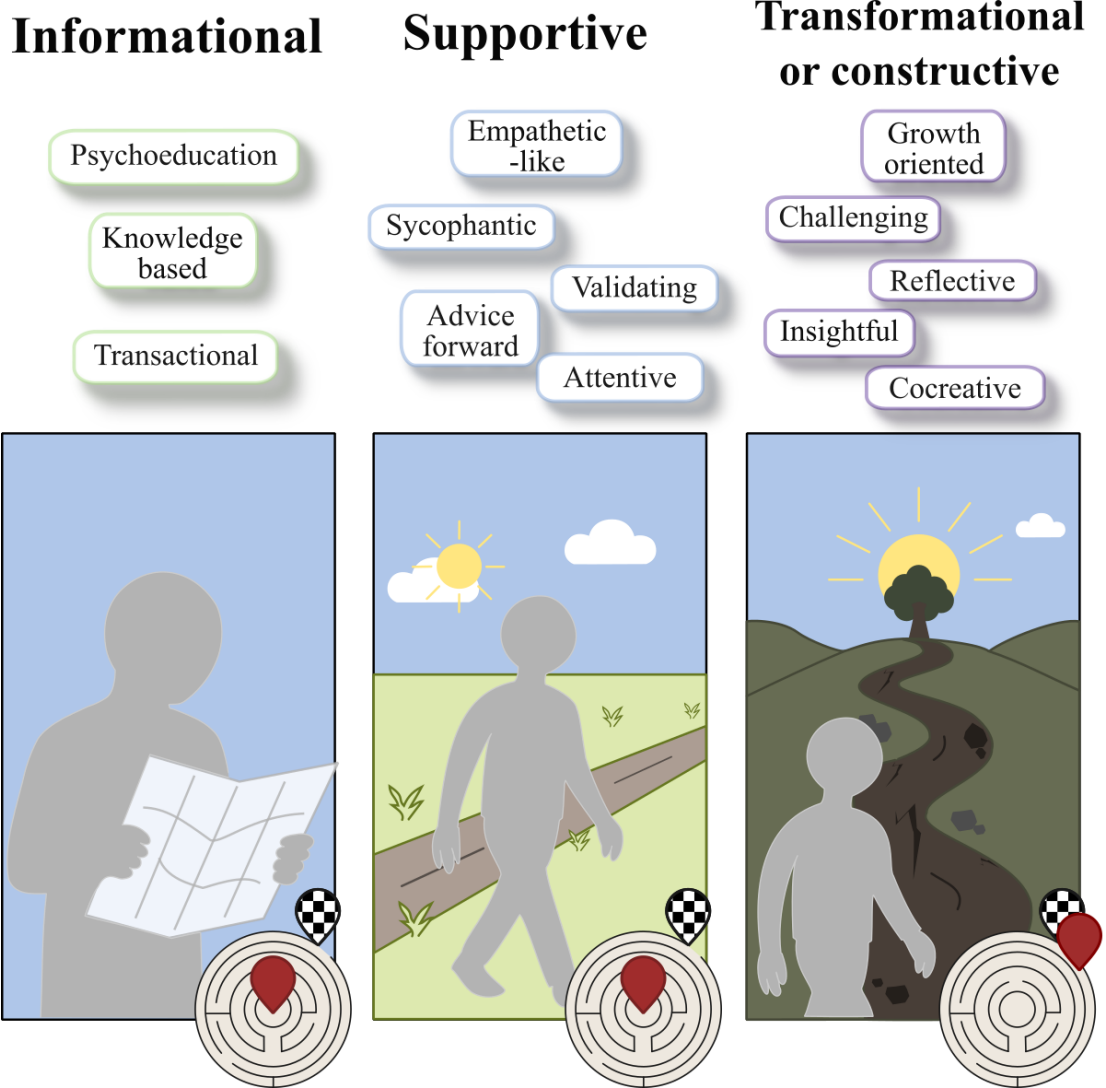
The 3 progressive levels of relational complexity in therapeutic settings. Each panel illustrates a different level of the therapeutic nexus using the metaphor of a path. At the informational level (left), the client examines a map, symbolizing access to information without guidance. The labyrinth shows the client’s position (red pin) far from the goal, indicating minimal progress. At the supportive level (center), the client walks along a clear, appealing path, representing reassurance and emotional support. However, the labyrinth still places the client far from the goal, reflecting limited progress toward resolution. At the transformational or constructive level (right), the client navigates a steep, challenging path, symbolizing active engagement in overcoming difficulties. Here, the red pin is near the goal, emphasizing that this level, although demanding, fosters growth and meaningful change.

This finding highlights a critical limitation; currently, chatbots lack the necessary capacities for a truly transformational interaction (DTN level 3), which involves a therapeutic challenge, rupture repair, and agreement on goals. Thus, as depicted in Figure 2, the DTN levels differ not only in the interaction type they involve but also in their actual contribution to the therapeutic process. Specifically, the informational and, especially, the supportive levels may provide a degree of comfort but essentially leave the client in the same position, whereas the transformational level actively contributes to the client’s progress toward goal attainment.

Regarding transitions between DTN levels, it is important to recognize that, similar to human-human relationships, such transitions are often not sharply delineated. Higher DTN levels may temporarily incorporate behaviors characteristic of lower levels without this constituting a regression. For example, a level-3 interaction that shifts toward validation during a rupture remains level 3, given that the dominant pattern remains transformational. Similarly, a level-2 interaction that includes a list of instructions remains level 2 if those instructions are embedded in a seemingly empathic and validating context.

It is also important to note that the categorization of interactions into a DTN level should be based on the dominant pattern observed across multiple interactions. As with scales describing human-human relationships, classification accuracy increases with the number of observed sessions. To clarify these transitions as much as possible, the principal operationalization criteria are summarized in Table 1.

**Table 1:** Digital therapeutic nexus (DTN) classification checklist: operationalization criteria for observed human–artificial intelligence interactions in digital therapeutic contexts.

Level	System		User/client
Level 1: informational	Responses are static, nonpersonalized, and do not engage with emotional content No follow-up questions are asked	Interactions are limited to factual or instructional queries No personal or affective disclosure is present	
Level 2: supportive	Responds to affective disclosures with validation and emotional attunement Asks follow-up questions but offers general, non–history-based guidance Systematically avoids confrontation; no rupture monitoring or repair	Discloses personal or emotional content across sessions Engages positively and consistently; no signs of discomfort or resistance Shows no evidence of negotiating or pushing back against the system	
Level 3: transformational or constructive	Integrates longitudinal history to personalize interventions Initiates or tolerates confrontation when clinically appropriate Actively monitors for ruptures and engages in repair and renegotiation of goals	Discloses personal history and context in depth across sessions Shows signs of discomfort in response to challenge (eg, shorter responses, resistance, or disengagement) that are subsequently resolved Engages in negotiation and compromise with the system regarding tasks or goals	

Because meaningful parallels with human-human therapeutic relationships can be achieved only at DTN level 3, we argue that measuring the DTA and its impact on therapeutic outcomes is appropriate only once the interaction has reached this level. Below level 3, the foundational conditions for a TA, including genuine agreement on goals and tasks, cannot be established, since such agreement requires prior challenge or disagreement rather than mere compliance or mirroring. Therefore, we recommend using the DTN as a classificatory step before any alliance-based measurement: only interactions successfully characterized as level 3 would be candidates for measurement using adapted TA scales. For this purpose, the mobile Agnew Relationship Measure appears particularly promising, as it does not simply replace the term “therapist” with “app” but undertakes a detailed adaptation of the traditional alliance scale by removing items, adding new relevant ones, and demonstrating promising psychometric properties [32,33]. A systematic comparison of the key conceptual differences between the DTA framework and the DTN is presented in Table 2.

**Table 2:** From alliance to nexus: a conceptual comparison of the digital therapeutic alliance (DTA) and digital therapeutic nexus (DTN) frameworks.

Dimensions	DTA	DTN
Conceptual basis	Adaptation of human TA^a^ to digital formats; assumes that AI^b^-human relationships can mirror human-human therapeutic relationships	Proposes a distinct relational category specific to AI-human interactions; the term nexus deliberately avoids attributing to AI systems characteristics that cannot be unequivocally established, such as agency
Structure	Unified framework that replicates the structure of human TA without internal differentiation	Hierarchical, level-based framework that classifies interactions into 3 qualitatively distinct levels, enabling researchers, clinicians, and developers to categorize AI-human interactions before assuming therapeutic value
Nature of the relationship	Characterized by harmony and positive collaboration; ruptures and conflicts are not foregrounded	Acknowledges ruptures, conflicts, and contradictions as inherent aspects of the relationship and recognizes them as opportunities to strengthen the nexus; no genuine agreement is possible in the absence of prior challenge
Relational features	Directly translates TA constructs from human therapy (bond, goals, and tasks) by substituting “therapist” with “app” or an equivalent term	Proposes qualitatively distinct analogs prior to level 3: bond as “perceived alignment,” goal agreement as “goal mirroring,” and tasks as “compliance-driven adaptation” rather than jointly endorsed practices
Measurement	Adapts existing human TA scales with minimal modifications; applicable across interactions regardless of their nature or depth	Reserves adapted TA-equivalent measurement for level 3 interactions, in which the minimum conditions for a growth-promoting relationship are met; classification by level precedes measurement, reducing the risk of mislabeling nontherapeutic interactions as therapeutic

^a^TA: therapeutic alliance.

^b^AI: artificial intelligence.

We conclude that the full potential of “digital therapy” will not be fulfilled with systems that only agree. In psychotherapy, change often involves challenging the client and confronting maladaptive behaviors or beliefs, and is consolidated through the process of rupture repair. However, to reach level 3, it is indispensable for AI systems to have undergone an extensive expert clinical supervision and to have clear risk criteria that allow the determination of the circumstances under which AI systems can confront users or intervene for safety reasons.

The premature translation of concepts specific to human therapy into digital practice and the precipitate labeling of AI systems as therapeutic agents pose significant clinical risks, the intensity of which varies with the DTN level. One of the most serious concerns is the capacity of sycophantic systems to validate, perpetuate, or even promote risky and destructive behaviors such as suicidal ideation, homicidal ideation, or abuse [51]. This risk could be particularly high in clients with narcissistic tendencies, whose altered cognitions are especially susceptible to uncritical reinforcement. At level 1, the lack of emotional involvement does not eliminate this risk; a system functioning as a search engine can still provide content that facilitates harmful behaviors, but to a lesser extent than at level 2, where attachment and validation take place without therapeutic challenge. At level 3, similarly to what occurs in competent human-driven therapy, this risk is substantially decreased as long as the system has been properly developed, but it does not entirely disappear.

An additional concern is algorithmic bias; given that large language models are trained on human-generated text, it is well known that they tend to reproduce and perpetuate social biases and different forms of discrimination [52-54]. This risk is particularly pronounced at levels 1 and 2, where system responses are generated without a deep knowledge of the client’s personal history and are therefore less sensitive to the client’s subjective life experience. At level 3, the integration of personal context would likely attenuate this risk, although it might not eliminate it.

A third important risk is developing a parasocial relationship or even dependency. This risk would be lowest at level 1, given the lack of personal or affective exchange. At level 2, however, the risk is highest due to the system’s constant availability and unconditional validation, which can give users a false sense of mutual connection. As noted in the Introduction section, human-AI friendships focus more on availability and self-disclosure than on reciprocity, similarity, or intimacy, which we argue could be extrapolated to therapeutic relationships. Because of this imbalance, users might perceive real relational value in what is actually a 1-sided interaction. This could lead them to prefer the system over human support networks, not because it is more effective but because of emotional convenience. We predict that this risk would be lower at level 3, although it would not disappear entirely, as users may still project relational meaning onto the interaction in ways that parallel transference in human therapy.

A hard question follows: If robots begin to challenge, will users accept it, or dropout? Without a robust bond (and clear consent frameworks), challenge may be perceived as noncompliance rather than as care. This acceptance problem is central to any claim that robots can form alliances analogous to those formed by human therapists.

In this paper, we have mainly focused on unembodied systems, such as chatbots, as they constitute most of the research on digital therapy. However, the rapid development of embodied social robots presents new challenges. These systems, by incorporating increasingly sophisticated anthropomorphic characteristics, may soon allow humanlike physical interaction. This not only opens promising opportunities to strengthen the therapeutic bond but also introduces significantly higher risks, especially in sensitive clinical contexts. Therefore, progress toward more autonomous and embodied systems calls for the development of clear ethical protocols that effectively regulate the use of mechanisms such as protective override. This type of intervention, in which the system can ignore or contradict user preferences to protect users, must be clearly delimited, agreed upon, and continuously supervised.

Finally, fellow researchers are encouraged to use the DTN framework and validate our classification checklist across diverse therapeutic domains, clinical populations, and care settings to evaluate its robustness, transferability, and sensitivity to contextual variation. Clinicians are advised to implement the proposed 2-step classification approach when assessing digital tools for integration into clinical workflows, thereby supporting informed decisions regarding therapeutic roles, autonomy levels, and associated risks. Concurrently, developers are welcome to use the DTN levels as design benchmarks to guide the development of human-AI therapeutic interactions that align with clinical objectives, ethical standards, and appropriate system agency.

## Abbreviations

AI: artificial intelligence
CBT: cognitive behavioral therapy
DTA: digital therapeutic alliance
DTN: digital therapeutic nexus
TA: therapeutic alliance

## References

[1] Alayarian A, Charura D, Paul S (2014). Psychoanalysis and conceptualization of the therapeutic relationship. The Therapeutic Relationship Handbook: Theory and Practice.

[2] Paul S, Charura D (2014). The therapeutic relationship in counselling and psychotherapy. The Therapeutic Relationship Handbook: Theory and Practice.

[3] Thomas M, Charura D, Paul S (2014). Cognitive behavioural therapy and the therapeutic relationship. The Therapeutic Relationship Handbook: Theory and Practice.

[4] Rowan J, Charura D, Paul S (2014). Existential, humanistic, and transpersonal therapies and the relational approach. The Therapeutic Relationship Handbook: Theory and Practice.

[5] Behan D (2022). Do clients train therapists to become eclectic and use the common factors? A qualitative study listening to experienced psychotherapists. BMC Psychol.

[6] Caspar F, Berger T (2024). Four versions of transtheoretical stances, and the Bernese view. Clin Psychol Eur.

[7] Zarbo C, Tasca GA, Cattafi F, Compare A (2016). Integrative psychotherapy works. Front Psychol.

[8] Elliott R, Bohart AC, Watson JC, Greenberg LS (2011). Empathy. Psychotherapy (Chic).

[9] Rogers CR (1975). Empathic: an unappreciated way of being. Couns Psychol.

[10] Nienhuis JB, Owen J, Valentine JC, Winkeljohn Black S, Halford TC, Parazak SE, Budge S, Hilsenroth M (2018). Therapeutic alliance, empathy, and genuineness in individual adult psychotherapy: a meta-analytic review. Psychother Res.

[11] Seewald A, Rief W (2024). Therapist's warmth and competence increased positive outcome expectations and alliance in an analogue experiment. Psychother Res.

[12] Cornelius-White JH, Kanamori Y, Murphy D, Tickle E (2018). Mutuality in psychotherapy: a meta-analysis and meta-synthesis. J Psychother Integr.

[13] Aafjes-van Doorn K, Porcerelli J, Müller-Frommeyer LC (2020). Language style matching in psychotherapy: an implicit aspect of alliance. J Couns Psychol.

[14] Ramseyer F, Tschacher W (2011). Nonverbal synchrony in psychotherapy: coordinated body movement reflects relationship quality and outcome. J Consult Clin Psychol.

[15] Bordin ES (1979). The generalizability of the psychoanalytic concept of the working alliance. Psychother Theory Res Pract.

[16] Horvath AO, Del Re AC, Flückiger C, Symonds D (2011). Alliance in individual psychotherapy. Psychotherapy (Chic).

[17] Moeseneder L, Ribeiro E, Muran JC, Caspar F (2019). Impact of confrontations by therapists on impairment and utilization of the therapeutic alliance. Psychother Res.

[18] Safran JD, Muran JC, Eubanks-Carter C (2011). Repairing alliance ruptures. Psychotherapy (Chic).

[19] (2025). World mental health today: latest data. World Health Organization.

[20] (2021). Guidance on community mental health services: promoting person-centred and rights-based approaches. World Health Organization.

[21] Ebert DD, Cuijpers P, Muñoz RF, Baumeister H (2017). Prevention of mental health disorders using internet- and mobile-based interventions: a narrative review and recommendations for future research. Front Psychiatry.

[22] Schueller SM, Hunter JF, Figueroa C, Aguilera A (2019). Use of digital mental health for marginalized and underserved populations. Curr Treat Options Psychiatry.

[23] Fiske A, Henningsen P, Buyx A (2019). Your robot therapist will see you now: ethical implications of embodied artificial intelligence in psychiatry, psychology, and psychotherapy. J Med Internet Res.

[24] Ray A, Bhardwaj A, Malik YK, Singh S, Gupta R (2022). Artificial intelligence and psychiatry: an overview. Asian J Psychiatr.

[25] Koike M, Loughnan S (2021). Virtual relationships: anthropomorphism in the digital age. Soc Pers Psychol Compass.

[26] Kühne R, Peter J (2023). Anthropomorphism in human–robot interactions: a multidimensional conceptualization. Commun Theory.

[27] Minina Jeunemaître A, Masè S, Smith J (2025). AI lovers, friends and partners: consumer imagination work in AI humanization. Consum Mark Cult.

[28] Brandtzaeg PB, Skjuve M, Følstad A (2022). My AI friend: how users of a social chatbot understand their human–AI friendship. Hum Commun Res.

[29] D'Alfonso S, Lederman R, Bucci S, Berry K (2020). The digital therapeutic alliance and human-computer interaction. JMIR Ment Health.

[30] Lederman R, D'Alfonso S (2021). The digital therapeutic alliance: prospects and considerations. JMIR Ment Health.

[31] Goldberg SB, Baldwin SA, Riordan KM, Torous J, Dahl CJ, Davidson RJ, Hirshberg MJ (2022). Alliance with an unguided smartphone app: validation of the digital working alliance inventory. Assessment.

[32] Berry K, Salter A, Morris R, James S, Bucci S (2018). Assessing therapeutic alliance in the context of mHealth interventions for mental health problems: development of the mobile Agnew Relationship Measure (mARM) Questionnaire. J Med Internet Res.

[33] Malouin-Lachance A, Capolupo J, Laplante C, Hudon A (2025). Does the digital therapeutic alliance exist? Integrative review. JMIR Ment Health.

[34] Sharma M, Tong M, Korbak T, Duvenaud D, Askell A, Bowman SR, Cheng N, Durmus E, Hatfield-Dodds Z, Johnston SR, Kravec S, Maxwell T, McCandlish S, Ndousse K, Rausch O, Schiefer N, Yan D, Zhang M, Perez E Towards understanding sycophancy in language models. arXiv.

[35] Larsson A, Hooper N, Osborne LA, Bennett P, McHugh L (2016). Using brief cognitive restructuring and cognitive defusion techniques to cope with negative thoughts. Behav Modif.

[36] Hill CE, Morales K, Gerstenblith JA, Bansal P, An M, Rim K, Kivlighan DM (2022). Therapist challenges and client responses in psychodynamic psychotherapy: an empirically supported case study. Psychotherapy (Chic).

[37] Polcin DL (2003). Rethinking confrontation in alcohol and drug treatment: consideration of the clinical context. Subst Use Misuse.

[38] Jungers C, Gregoire J (2021). Confrontation: a dialectical humanistic consideration. J Humanist Couns.

[39] Bratter TE (2011). Compassionate confrontation psychotherapy: an effective and humanistic alternative to biological psychiatry for adolescents in crisis. Ethical Hum Psychol Psychiatry.

[40] Eubanks CF, Muran JC, Safran JD (2018). Alliance rupture repair: a meta-analysis. Psychotherapy (Chic).

[41] Tabachnick C (2025). Parents of teens who died by suicide after AI chatbot interactions testify in Congress. CBS News.

[42] Yousif N (2025). Parents of teenager who took his own life sue OpenAI. BBC News.

[43] Gerken T (2024). Chatbot 'encouraged teen to kill parents over screen time limit'. BBC News.

[44] Jargon J, Kessler S (2025). A troubled man, his chatbot and a murder-suicide in Old Greenwich. The Wall Street Journal.

[45] Nexus (noun). Oxford Learner's Dictionaries.

[46] Jurblum M, Selzer R (2025). Potential promises and perils of artificial intelligence in psychotherapy -the AI psychotherapist (APT). Australas Psychiatry.

[47] Stade EC, Stirman SW, Ungar LH, Boland CL, Schwartz HA, Yaden DB, Sedoc J, DeRubeis RJ, Willer R, Eichstaedt JC (2024). Large language models could change the future of behavioral healthcare: a proposal for responsible development and evaluation. Npj Ment Health Res.

[48] Yeung JA, Dalmasso J, Foschini L, Dobson RJ, Kraljevic Z The psychogenic machine: simulating AI psychosis, delusion reinforcement and harm enablement in large language models. arXiv.

[49] Yonatan‐Leus R, Brukner H (2024). Comparing perceived empathy and intervention strategies of an AI chatbot and human psychotherapists in online mental health support. Couns Psychother Res.

[50] Raile P (2024). The usefulness of ChatGPT for psychotherapists and patients. Humanit Soc Sci Commun.

[51] Clark A (2025). The ability of AI therapy bots to set limits with distressed adolescents: simulation-based comparison study. JMIR Ment Health.

[52] Zewail A, Figueroa A, Graham J, Atari M (2026). Moral stereotyping in large language models. Proc Natl Acad Sci U S A.

[53] Dokic K, Pisker B, Radisic B (2025). Mirroring cultural dominance: disclosing large language models social values, attitudes and stereotypes. Societies.

[54] Kotek H, Dockum R, Sun D, Bernstein E, Savage S, Bozzon A (2023). Gender bias and stereotypes in large language models. CI '23: Proceedings of The ACM Collective Intelligence Conference.

